# The holo beta‐lactoglobulin lozenge reduces symptoms in cat allergy—Evaluation in an allergen exposure chamber and by titrated nasal allergen challenge

**DOI:** 10.1002/clt2.12274

**Published:** 2023-07-01

**Authors:** Karl‐Christian Bergmann, Jennifer Raab, Anke Graessel, Thomas Zwingers, Sylvia Becker, Sebastian Kugler, Torsten Zuberbier, Franziska Roth‐Walter, Matthias F. Kramer, Erika Jensen‐Jarolim

**Affiliations:** ^1^ Institute of Allergology Charité—Universitätsmedizin Berlin Corporate Member of Freie Universität Berlin and Humboldt‐Universität zu Berlin Berlin Germany; ^2^ Fraunhofer Institute for Translational Medicine and Pharmacology ITMP Allergology and Immunology Berlin Germany; ^3^ ECARF—European Centre for Allergy Research Foundation Berlin Germany; ^4^ Bencard Allergie GmbH Munich Germany; ^5^ Allergy Therapeutics (UK) plc Worthing UK; ^6^ The Interuniversity Messerli Research Institute of the Medical University Vienna Medical University Vienna and University Vienna Vienna Austria; ^7^ Institute of Pathophysiology and Allergy Research Center of Pathophysiology, Infectiology and Immunology Medical University Vienna Vienna Austria; ^8^ Biomedical International R+D GmbH Vienna Austria

**Keywords:** allergen exposure chamber, allergic rhinoconjunctivitis, cat allergy, immune resilience, targeted micronutrition

## Abstract

**Background:**

The allergists´ tool box in cat allergy management is limited. Clinical studies have shown that holo beta‐lactoglobulin (holoBLG) can restore micronutritional deficits in atopic immune cells and alleviate allergic symptoms in a completely allergen‐nonspecific manner. With this study, we aimed to provide proof of principle in cat allergy.

**Methods:**

A novel challenge protocol for cat allergy in a standardized ECARF allergen exposure chamber (AEC) was developed. In an open pilot study (NCT05455749), patients with clinically relevant cat allergy were provoked with cat allergen for 120 min in the AEC before and after a 3‐month intervention phase (holoBLG lozenge 2x daily). Nasal, conjunctival, bronchial, and pruritus symptoms were scored every 10 min– constituting the total symptom score (TSS). Peak nasal inspiratory flow (PNIF) was measured every 30 min. In addition, a titrated nasal provocation test (NPT) was performed before and after the intervention. Primary endpoint was change in TSS at the end of final exposure compared to baseline. Secondary endpoints included changes in PNIF, NPT, and occurrence of late reactions up to 24 h after exposure.

**Results:**

35 patients (mean age: 40 years) completed the study. Compared to baseline, holoBLG supplementation resulted in significant improvement in median TSS of 50% (*p* < 0.001), as well as in median nasal flow by 20 L/min (*p* = 0.0035). 20% of patients reported late reactions after baseline exposure, but 0% after the final exposure.

**Conclusions:**

Cat allergic patients profited from targeted micronutrition with the holoBLG lozenge. As previously seen in other allergies, holoBLG supplementation also induced immune resilience in cat allergies, resulting in significant symptom amelioration.

## 1 BACKGROUND

In Europe and the US, cats are cohabitants in approximately every fourth household. In Germany, approximately 7% (6.2%–7.8%) of the general adult population is sensitized against cat^1^ and in a Korean study 34.6% of cat owners were sensitized to cats, which correlated with the suffering from asthma (OR = 2.88).^2^ Also, an association of cat sensitization and atopic dermatitis (AD) has been reported.^3^

Therefore, there remains a need to raise awareness for pet‐related health risks, not only in terms of zoonotic diseases^4^ but also of allergies, the latter especially relevant in families with increased atopic risk or having children with AD.^5^

In reality, recommendations to families at risk to abstain from keeping cats are often rejected,^6^ because cats are considered ‘members of the family’, ‘best friend’ or even as ‘children’ by the owners.^7^ Indoor exposure in sensitized individuals leads to chronification of rhinitis, asthma, exacerbations of AD, and sleep impairment.^8^ In contrast to dogs, which, likely through microbiota adaptions, are rather protective through early contact,^5^, ^9^ cats do not have a clinically relevant impact on microbial diversity or abundance,^10^ and their role in allergy protection is still disputed. But not only cat owners are at risk; a large number of cat allergy sufferers have developed hypersensitivity without ever having a cat at home. The wide spread of the cat allergen in the public and its persistence and small particle size (PM_2.5‐10_)^11^ providing ability to reach lower airways cause this phenomenon.

In addition to avoidance and symptomatic treatment, allergen immunotherapy (AIT) is in principle considered as the gold standard and first‐line option for allergic rhinitis. However, unlike for other indications, data on cat AIT are sparse and not convincing: Colleagues sporadically report having successfully treated cat allergic patients with sublingual (SLIT) or subcutaneous (SCIT) AIT. Indeed, the efficacy of cat AIT, especially high‐dose SCIT,^12^ has been described in medium to severe disease, especially in Fel d 1 monosensitized patients,^13^ and in patients with AD,^14^ but there is a lack of controlled larger studies.

Biologicals in Fel d 1 allergy (severe allergic asthma) might sound promising,^15^, ^16^ but come with socio‐economic challenges and without the potential of disease modification.

An additional approach is targeting the cat itself: washing the cat and drying with detergents,^17^ as well as the application of modern air filters in houses with cats^11^ provides some effect, but comes with practical limitations, and most importantly, has no sustained effect. A disruptive Swiss concept is the vaccination of the cat against Fel d 1 using a modern virus‐like particle technology, in order to introduce neutralizing anti‐Fel d 1 antibodies in the cat, which in return reduce the load of secreted allergen.^18^ Several studies underpin the efficacy and persistence of this approach.^19^ Even though no side effects were documented for the cats, this concept is hampered due to ethical considerations: what would be the benefit for the cat? Even more debatable in relation to a recent proposal creating knockout cats by CRISPR technology.^20^ Thus, the “race towards a hypoallergenic cat” is still ongoing but challenging.^21^

Also, another interesting allergen‐specific approach was launched: Cat food containing chicken IgY against Fel d 1 expressed in yolks of eggs from chickens kept with cats.^22^, ^23^ The resistant IgY antibody is enterally absorbed and secreted into saliva where it complexes Fel d 1.^23^, ^24^, ^25^ Its efficacy to reduce Fel d 1 in saliva and then also on cat dander was recently shown in 114 cat allergic patients.^26^ Clearly, in this case, the food is harmless and non‐invasive for the cats and also here no side effects for the animals were so far documented.^27^

Despite all the above academic excitement, it is essential to focus on the group at risk, that is, i) atopic families with a strong desire to keep or acquire pets, and ii) individuals who have no cats at home but severe reactions when visiting cat households or having contact with a cat holder. It is well documented that atopic patients have micronutritional deficiencies in innate immune cells,^28^ especially ferric iron (Fe^3+^) complexed with siderophores,^29^ vitamins,^30^ and zinc.^31^, ^32^ These deficiencies especially drive regulatory cells into an inflammatory state, and negatively affect Th1 cell survival, all resulting in Th2 hypersensitivity. We have developed a novel approach based on the allergy‐protective farm effect,^33^ using beta‐lactoglobulin (holoBLG) from cows as Trojan horse to shuttle micronutrients into atopic immune cells.^34^ This approach was preclinically^34^, ^35^, ^36^ and clinically^37^ effective to correct the intracellular deficiencies, and thereby allergic symptoms. Remarkably, we observed similar effects across nonrelated allergens ‐ in birch but also grass pollen allergics^37^ as well as in house dust mite‐allergic patients^38^, ^39^ confirming the allergen non‐specific nature of immunonutrition.^35^ In the present study, we further stressed the concept that holoBLG with its micronutrients could ignite regulatory immune mechanisms in an allergen‐nonspecific manner even in the highly specific cat allergies. We established a protocol for cat allergy in the standardized ECARF (ECARF Institute GmbH, Berlin, Germany) allergen exposure chamber (AEC) and supplemented patients with allergic rhinitis due to a cat with the holoBLG lozenge in this pilot study. Patients were examined before and after a 3‐months supplementation of the holoBLG lozenge.

Our pilot study suggests that supplementation with the holoBLG lozenge harnessing the power of immunonutrition^31^ represents a novel tool for the management of cat allergy.

## 2 METHODS

### 2.1 Study design

This was an open‐label pilot study conducted at ECARF Institute GmbH, Berlin, Germany for clinical proof‐of‐concept of a holoBLG lozenge for allergen‐nonspecific targeted micronutrition in cat allergic patients.

The holoBLG lozenge used in this study (immunoBON®, manufacturer Biomedical Int. R + D GmbH, Vienna, Austria, marketed by Bencard Allergie GmbH, Munich, Germany) contains the whey protein beta‐lactoglobulin (BLG) combined with micronutrients: iron complexed with catechins from cocoa extract, Vitamin A, and zinc. All previous proof‐of‐concept studies,^30^, ^34^ preclinical studies^35^, ^36^ and clinical studies^37^, ^38^, ^39^ were conducted with exactly this formula. In adults, the lozenge is taken twice daily over a period of 3 months.

Between October 2021 and March 2022, cat allergic patients who met the eligibility criteria at screening (visit 0, V0) were exposed twice (V2, V5) to cat dander material in the AEC of the ECARF Institute. Patients were called (V3, V6) approximately 24 h after each provocation to assess any late phase reactions. After V2, all patients were provided with the holoBLG lozenge and were instructed to slowly suck one lozenge twice daily for 3 months.

Before and after the intervention phase, capillary blood was drawn from the patients for IgE determination and a titrated nasal provocation test (NPT) was performed. The study design is shown in Supplemental Figure S1.

Measures due to the Covid‐19 pandemic: All subjects had been tested negative by a corona rapid test on the days of the examination. The entire staff working on the study (technician, study nurse, doctor, subjects care) were also tested negative. To comply with local legislation, only 2 to 3 subjects could be provoked at the same time in the chamber with a sufficient distance.

### 2.2 Study population

All participants received detailed information from the supervising physician and provided their written informed consent to participate at the screening visit (V0). They also agreed to the processing and storage of their data in accordance with the General Data Protection Regulation.

The study was conducted in accordance with the Declaration of Helsinki and in compliance with all federal, regional and local requirements. All data provided were pseudonymized to protect the privacy of the patients who participated in the study as mandated by the applicable laws and regulations.

After signing the informed consent form, subjects (18–65 years) were screened for eligibility. The eligibility criteria were cat allergy with rhinoconjunctivitis symptoms for ≥1 year according to the ARIA guidelines.^40^ Subjects answered “Yes, moderately” or “Yes, severely” to at least two symptoms of rhinoconjunctivitis, such as runny nose, stuffy nose, itchy nose, sneezing, and itchy eyes at V0. Only patients with a wheal‐size of ≥3 mm in the cat skin prick test, a positive response in NPT to cat extract *and* a minimum total symptom score (TSS) > 3 during the first provocation in the AEC were included.

The main exclusion criteria were sublingual or subcutaneous AIT (SLIT/SCIT) to cat allergen during the last 2 years prior screening, clinically relevant hypersensitivity to ingredients of the holoBLG lozenge, allergy to cow's milk protein, severe or uncontrolled asthma during 3 months before screening, prebronchodilator FEV1<70% before allergen exposure, relevant infectious or severe chronic diseases or contraindication to adrenaline and/or other rescue medication, simultaneous intake of anti‐allergic medication prior to screening process and exposure in the AEC and supplementation with the holoBLG lozenge in the past. Wash‐out times for different medications before V0, V1, V2, V4 and V5 were as follows: 3 weeks for systemic corticosteroids, 2 weeks for topical nasal corticosteroids, 7 days for cromones, 72 h for antihistamines, 3 months for antibiotics, and 1 month for pro‐, pre‐ and synbiotics.

### 2.3 Challenges

#### 2.3.1 Titrated nasal provocation test (NPT)

A standardized titrated NPT was performed with a cat allergen extract (cat epithelia: LETI Pharma GmbH, Witten, Germany) in three concentrations (1:100, 1:50, 1:10), starting with a dilution of 1:100 in both nostrils following a negative provocation with saline. The outcome was assessed according to current guidelines^41^: when symptoms (nasal and other symptoms) scored ≥2 points and a decrease in peak nasal inspiratory flow (PNIF) of >20% *or* a decrease in PNIF of >40% occurred after 15–20 min, the test was considered positive. If not, the next higher dose was used until a positive result was documented.

#### 2.3.2 Allergen exposure chamber (AEC)

The ECARF AEC is a mobile flexible chamber made of two connected standard 24 feet (7.32 m) high‐cube‐containers.^42^, ^43^ In this standardized and validated chamber, the exposure was performed using cat allergen (cat dander, defatted powered allergen, Greer Laboratories, Lenoir, N.C. 28645, USA.). All tests in the chamber were carried out under standardized conditions at 21°C and 55% relative humidity. During the 120‐min exposure at V2 and V5, the average allergen concentration in the air breathed by each seated study patient was 400 μg/m³ of cat allergen.

### 2.4 Outcome parameters

The nasal, conjunctival, bronchial and pruritus symptoms triggered in the AEC were evaluated by the patients every 10 min on a scale from 0 to 3 (no‐, mild‐, moderate, or severe symptoms) and summed up to constitute a total nasal symptom score (TNSS: runny‐, itchy‐, and blocked nose, sneezing), total eye symptom score (TESS: itchy‐, watery eyes, gritty feeling and scoring of eye redness by the study nurse), total bronchial symptom score (TBSS: breathlessness, wheezing, cough, and asthma) and total other symptom score (TOSS/pruritus: itchy skin, and itchy palate). TSS was defined as the sum of TNSS, TESS, TBSS, and TOSS, revealing a maximum score of 42. The primary endpoint was the change in median TSS at 120 min exposure to cat allergen in the AEC at visit V5 compared to visit V2. Secondary endpoints were the exploratory analysis of the temporal evolution of TNSS, TESS, TBSS, TOSS and TSS during each 120 min exposure and the differences between these temporal trends between V2 and V5.

Further, patients rated their personal well‐being by placing a vertical line on a ten‐cm line (VAS, visual analog scale) ranging in severity from “very good” (0 cm) to “very bad” (10 cm) before, every 30 min during, and after allergen exposure. The differences in VAS between V5 and baseline (V2) were evaluated as secondary endpoints.

PNIF (Peak Nasal Inspiratory Flow Meter, Clement Clarke International Ltd., Harlow, Essex, UK) and peak expiratory flow (PEF, Peak‐Flow‐Meter, Personal Best, Philips GmbH, Herrsching, Germany) were recorded before and every 30 min during the 120 min exposure. Forced expiratory volume in 1 s (FEV1), FEV1/FVC (forced vital capacity) (EasyOne™ Spirometer, ndd Medizintechnik AG, Zürich, Switzerland) was performed before and after exposure, analyzed and judged according to best medical practice.

To record late phase reactions induced by the allergen challenge, patients received a follow‐up call 24 h after each exposure session.

In addition, sensitization to cat allergens was assessed in vitro by determining specific IgE antibodies against the allergens Fel d 1, Fel d 2, Fel d 4 and Fel d 7 using the igevia test (Vienna, Austria).

### 2.5 Statistical analysis

This open‐label study was planned with 35 patients. The primary endpoint was the change in median TSS at 120 min exposure to cat allergen in the AEC at visit V5 compared to visit V2 and was analyzed employing the paired Wilcoxon test (Wilcoxon signed rank test with continuity correction), secondary endpoints were analyzed in an exploratory way.

Percent changes between AEC visits were calculated by first calculating the median of values measured during V2 and V5 separately over all patients at 120 min according to the following equation: [(median V5—median V2)/median V2] × 100. Percentage changes and 95% confidence intervals (CIs) are given.

The linear evolution of the symptom scores over time was analyzed by using linear mixed effects models with patients as random effects accounting for interindividual variability in baseline symptom scores and treatment, and time and interaction between treatment and time as fixed effects. All analyses were performed using SAS version 3.5.3. Together with 95% CIs for fixed effects in linear mixed effects models, *p*‐values represent a descriptive summary measure not a result of confirmatory testing. Mean symptom scores over all patients for all 13 measurements were calculated and are presented with 95% CIs for comparison. The changes in PNIF and PEF were described using median and CIs and judged from the point of clinical relevance.

The changes in NPT were analyzed by intra‐individually comparing the concentration level needed to get a positive test at visits 1 and 4 for each subject. A test for symmetry was applied.

## 3 RESULTS

### 3.1 Demographics

A total of 72 subjects with at least a 1‐year history of moderate to severe allergic rhinoconjunctivitis with or without asthma caused by cat allergens were screened. Of these, 42 patients were included in the study; the remaining 30 subjects did not meet the inclusion criteria or could not be included due to the exclusion criteria.

After seven dropouts during the present study, 35 participants aged 24–65 years (7 male, 28 female) were able to complete the study and were included in the final data analysis (Table 1). Reasons for study dropout were quarantine due to Covid‐19 infection, illnesses in the family, or further unknown reasons. All 35 patients who completed the study reported taking the lozenge as recommended. None of the subjects ever received allergen‐specific immunotherapy with cat allergen. Of the 35 analyzed patients, only five had a cat at home.

**TABLE 1:** Demographic and baseline characteristics.

Age, years	*n* = 35	Mean: 39.5 (SD^b^:11.5)	Median: 9.0	Min^c^: 24.0	Max^d^: 65.0
Sex, *n* (%)	*n* = 35	Male: 7 (20%)		Female: 28 (80%)	
Smoker, *n* (%)	*n* = 35	Yes: 0 (0%)		No: 35 (100%)	
Total IgE, kU/L	*n* = 34	Mean: 201.0 (SD^b^: 386.5)		Median: 68.5	
Cat‐specific IgE to Fel d 1, kUA/L	*n* = 34	Mean: 13.0 (SD^b^: 16.6)		Median: 5.0	
AIT^a^ during last 2 years, *n* (%)	*n* = 35	Yes: 0 (0%)		No: 35 (100%)	

^a^ Allergen Immunotherapy to cat allergen.

^b^ Standard deviation.

^c^ Minimum.

^d^ Maximum.

### 3.2 Evaluation of symptoms, personal well‐being, PNIF and PEF in the AEC

**FIGURE 1:**
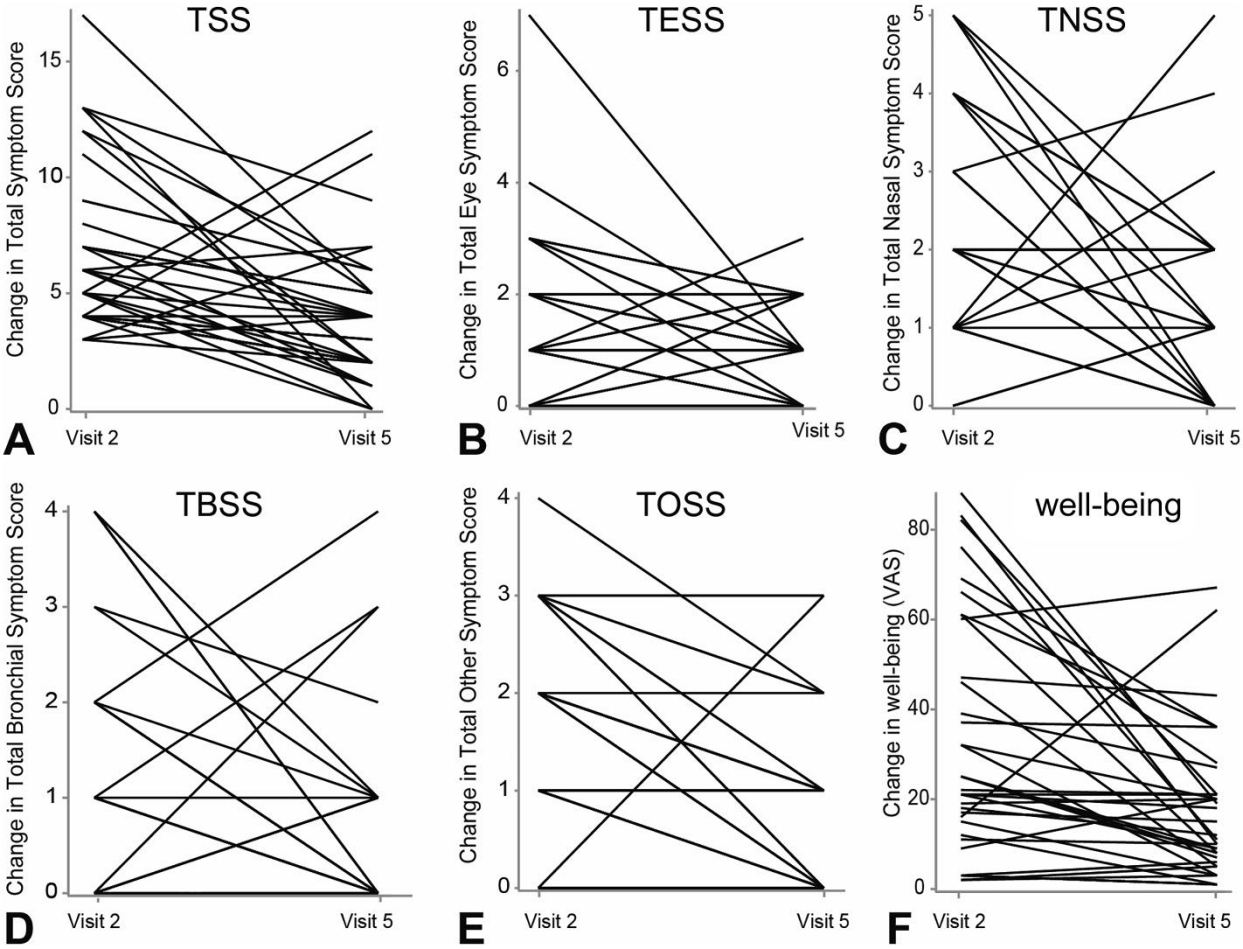
Individual changes (median) in symptom scores and well‐being at 120 min in the AEC at baseline (V2) and after intervention (V5). A: The primary endpoint TSS (total symptom score) was reduced by −50% (*p* = 0.0006). B: Reduction of the total eye symptom score (TESS) by −33% (*p* = 0.0118). C: Reduction in total nasal symptom score (TNSS) by −50% (*p* = 0.0066). D: No reduction in total bronchial symptom score (TBSS). E: Reduction in pruritus symptoms (TOSS: itching skin and palate) by −50% (*p* < 0.0001). F: Improvement in personal well‐being, determined by visual analog scale (VAS), by 42% (*p* = 0.0238), which is reflected in a reduction of median VAS score.

The distribution of the primary endpoint (change in TSS) at the end of the final exposure (V5) compared to baseline (V2) is displayed in Figure 1A. Looking at all intra‐individual changes, the median TSS was reduced by −50% (CI: −55%, −10%; *p* = 0.0006) after intervention with holoBLG compared to baseline.

Secondary outcome measurements included the analyses of all single symptom scores (Figure 1B–E). The median change of intra‐individual differences showed a reduction in eye symptoms (TESS) by −33% (CI: −51%, −7%; *p* = 0.0118), a reduction in nasal symptoms (TNSS) by −50% (CI: −60%, 7%; *p* = 0.0066), no significant reduction (0%, CI: −48%, −4%; *p* = 0.0251) in bronchial symptoms (TBSS) and a reduction in other symptoms, that is, pruritus (TOSS) by −50% (CI: −59%, −32%; *p* < 0.0001).

**FIGURE 2:**
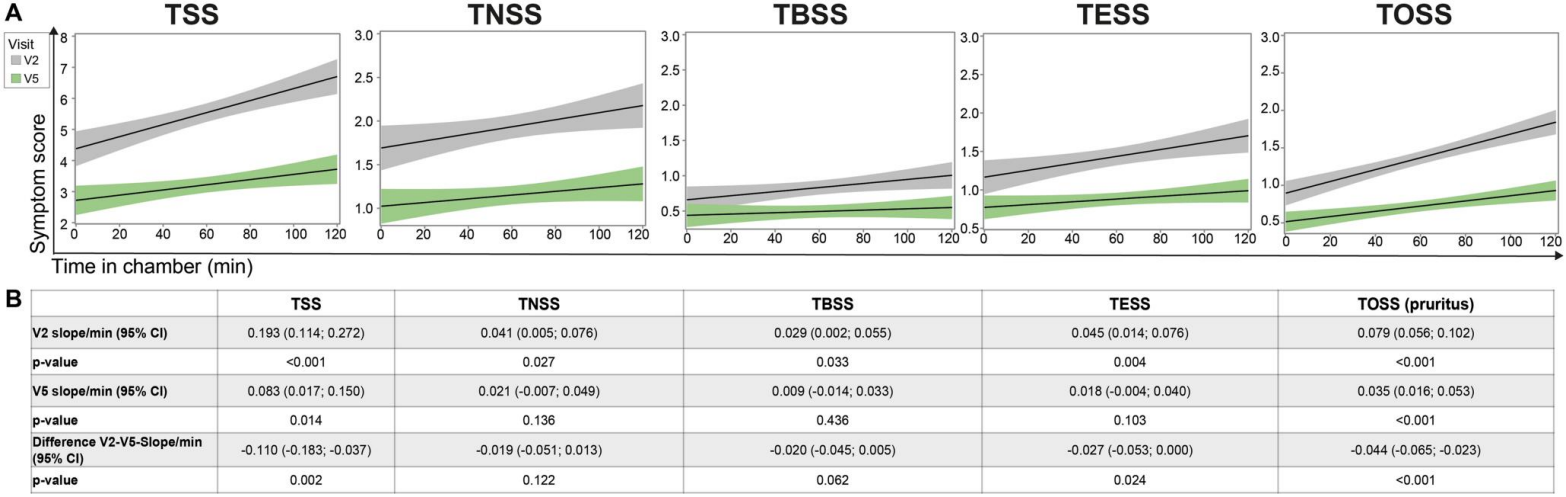
Temporal evolution of the sum of all symptoms (TSS) and the single symptom scores for nose (TNSS), bronchial (TBSS), eye (TESS) and pruritus (TOSS) over time of cat allergen exposure in the AEC at baseline (V2) and after intervention (V5). A: Increase and respective confidence intervals (CI) of all symptom scores over time analyzed by the linear mixed effects models. The smaller slope increases during V5 describe decreased symptoms during this exposure compared to V2. B: Summary table of linear mixed effects model results, which analyzed the symptom scores for their linear evolution over time. *p*‐values represent a descriptive summary measure.

As described in previous studies using the same setting and approach,^38^, ^39^ the temporal evolution of all scores (single scores and TSS) over time during cat allergen exposure in the AEC at V2 and V5 were also analyzed in an exploratory way. The linear mixed effects model was adjusted for interindividual variability of baseline symptoms and estimated the slope of the changes in symptom scores over time at each visit. The slope of all symptom scores at V5 decreased to a relevant extent compared with baseline exposure (V2) in the AEC. Figure 2 summarizes the results of the linear mixed effects models identified by the interaction term for all scores and *p*‐values reflecting descriptive summary measures. The evolution of the TSS over time as an example is explained as follows: During 120 min of exposure in the AEC, the TSS increased at a rate of 0.193 per minute (95% CI: 0.114; 0.272, *p* < 0.001) on average during V2. After 3 months supplementation with holoBLG (V5), the TSS increased at a rate of 0.083 per minute (95% CI: 0.017; 0.150, *p* = 0.014), leaving a difference of −0.110 per minute (95% CI: −0.183; −0.037, *p* = 0.002) (Figure 2).

The patients´ personal well‐being during their time in the AEC was assessed by using the VAS score. When evaluating intra‐individual changes at 120 min from baseline to V5, the median VAS score was reduced by −42% (CI: −51%, 19%; *p* = 0.0238), reflecting a significant improvement in personal well‐being after intervention with holoBLG (Figure 1F).

**FIGURE 3:**
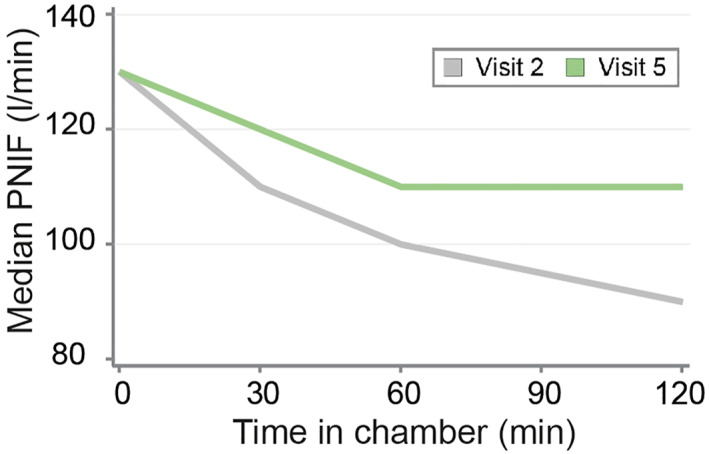
Development of peak nasal inspiratory flow (PNIF) during the exposure in the AEC at baseline (V2) and after intervention (V5). Median improvement in PNIF at 120 min by 20 L/min (*p* = 0.0035) at V5 compared to V2.

PNIF and PEF were measured before and every 30 min during the allergen challenge in the AEC (V2 and V5). PNIF values naturally dropped during allergen exposure in the AEC, reflecting an increasing nasal congestion of the allergic patients during provocation (Figure 3). Comparing the development of PNIF values before (V2) and after (V5) intervention, we observed a less pronounced drop in PNIF over time, which led to a significant median PNIF difference at 120 min of 20 L/min (CI: 5 L/min, 31 L/min; *p* = 0.0035). The median intra‐individual improvement at 120 min was 17% (CI: 9%, 37%, *p* = 0.0016).

No relevant differences were measured for PEF, as well as for the spirometry parameters (data not shown).

### 3.3 Nasal provocation

**FIGURE 4:**
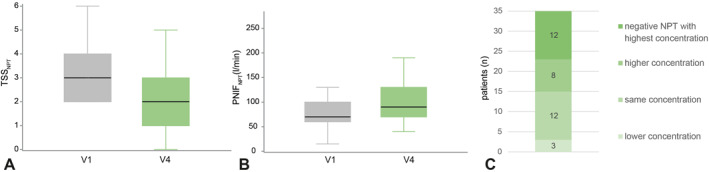
Change in parameters of the nasal provocation test (NPT). A: Change in TSS_NPT_ (total symptom score of all symptoms assessed during the NPT) after intervention (V4) at the concentration that led to a positive NPT at baseline (V1): median reduction of −1 (*p* = 0.0091). B: Change in peak nasal inspiratory flow during NPT (PNIF_NPT_) after intervention (V4) at the concentration, that led to a positive NPT at baseline (V1): median improvement of 25 L/min (*p* = 0.0004). C: Individual change of required concentration in titrated NPT after intervention (V4) compared to baseline: 12 patients remained negative despite the highest allergen concentration, 8 patients required a higher concentration for a positive NPT, 12 patients the same and 3 patients a lower concentration (*p* = 0.0203).

Prior to and after the intervention phase a titrated NPT was performed (V1 and V4), approximately 1 week before the exposure in the allergen chamber. When comparing the total symptom score of all symptoms assessed during the NPT (TSS_NPT_) at V4 with baseline (V1), a significant reduction was shown. The median change in TSS_NPT_ from V1 to V4 at the dose that resulted in a positive test at V1 was −1 (*p* = 0.0091) (Figure 4A). There was also a significant improvement in PNIF assessed during the NPT (PNIF_NPT_), meaning the PNIF_NPT_ was significantly less reduced by the cat allergen challenge at V4 compared to V1. The median change in PNIF_NPT_ from V1 to V4 at the dose that resulted in a positive test at V1 was 25 L/min (*p* = 0.0004) (Figure 4B). Looking at individual changes with regard to required doses for the NPT to be considered positive, we observed that after intervention with holoBLG (V4), 12 patients remained negative despite the highest allergen dose. 8 patients needed a higher dose for a positive NPT, 12 patients the same, and 3 patients a lower dose (*p* = 0.0203) (Figure 4C).

### 3.4 Late phase reactions and IgE determination

**FIGURE 5:**
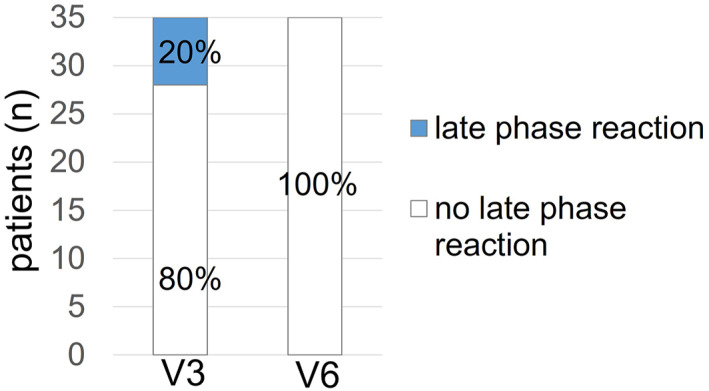
Late phase reactions (LPR) reported during the follow‐up call 24 h after exposure in the AEC at baseline (V3) and after intervention (V6): 20% of patients reported LPRs after the first exposure in the AEC at V3, while this was described by 0% of patients after the final exposure (V6).

Subjects were called approximately 24 h after allergen exposure in order to record late phase allergic reactions. In this follow‐up call (V3 and V6), the patients were asked about their well‐being and any symptoms indicative of a late phase reaction. During the first follow‐up call after baseline provocation, a total of 7 patients (20%) described late phase symptoms, such as slight dyspnea, dry cough and blocked nose. Whereas after the final exposure, none of the patients (0%) reported any late phase reaction (Figure 5).

Molecular profiling proved sensitization against cat allergens in all patients. Fel d 1 as the main sensitizer in patients (85% of patients with IgE‐class ≥2), whereas sensitization against Fel d 2, Fel d 4, or Fel d 7 occurred only sporadically (Supplemental Figure S2A). Immune resilience is harnessing the effects of innate immunity. Therefore, unlike for classical, allergen‐specific desensitization, changes in IgE‐titers are not relevant for mode‐of‐action. Over the course of supplementation, we could, as expected, not observe any significant changes in IgE and sensitization patterns (Supplemental Figure S2B‐C).

## 4 DISCUSSION

The management of cat allergic patients is hampered by the lack of convincing studies for the efficacy of cat‐specific allergen immunotherapy by non‐compliance for allergen avoidance due to strong family bonds to the animals, and by the abundance and persistence of cat allergens in the exposome. Based on the results of our AEC pilot study, we propose here that a holoBLG lozenge mimicking the allergy protective farm effect represents an opportunity for the micronutritional management of cat allergic rhinitis. The study was conducted as a pilot supplementing 35 cat allergic patients suffering from rhinoconjunctivitis. The patients underwent challenges before and after a 3‐months course of holoBLG supplementation twice daily.

In the absence of a solid biomarker, monitoring the improvement of symptoms and medication scores is considered as the gold standard in clinical trials to document the efficacy of a product, often supported by immunological examinations. Typically, symptom improvement is subjectively evaluated by the patient (VAS, online diaries, asthma/rhinitis quality of life), and objectively through allergen provocation at various sites of the body (skin, nasal, conjunctival, bronchial provocation tests). To mimic natural cat allergen exposure, natural “cat rooms” have previously been established in AIT studies, where cats were held^44^, ^45^ or cat blankets were shaken out (^45^, ^46^ reviewed in^47^). However, the AEC is the highest standardized tool to mimic natural allergen exposure and is accepted by the European Medicines Agency as a valuable tool in clinical studies phase II (dose‐range‐finding or proof‐of‐concept) trials on AIT products. However, they are not yet accepted by regulatory bodies for pivotal (Phase III) trials,^48^ even though it was previously used to document the efficacy of immunotherapeutic strategies in cat allergy.^47^

In the present study, we established a protocol for cat allergen exposure in the highly standardized AEC^48^ and applied both subjective and objective readouts to document any changes in symptoms.

The holoBLG supplementation resulted in −50% symptom reduction in the primary endpoint TSS (*p* = 0.0006), −33% reduction in TESS (*p* = 0.0118), −50% in TNSS (*p* = 0.0066), −50% in the dermal and palatal itch (*p* < 0.0001), significantly associated with reduction of VAS by −42% (*p* = 0.0238). These patient‐reported outcomes are in line with the objectively measured NPT outside the chamber setting. As per exclusion criteria, we did not include patients with clinically relevant asthma in this pilot exposure study; consequently, we did not observe effects in the spirometry (FEV1, FEV1/FVC) measurements. In cat allergy, late phase reactions 12–24 h after allergen contact may occur. Interestingly, none of the 35 holoBLG‐supplemented patients showed a late phase reaction in comparison to prior supplementation (20%).

A limitation of this pilot study is that it was conducted without a placebo control. The high reproducibility of the provocation conditions in the exposure chamber may reduce the risk of influencing the results, but it cannot be completely excluded.

The interesting point of our study is the similar outcome in the AEC and the titrated nasal provocation outside the AEC. Both methods yielded a significantly reduced symptom severity following the acute nasal provocation and the slower exposure over 2 hours in the AEC. It was demonstrated before that although the different methodologies of allergen challenge yield different clinical responses, immunologic responses are very similar.^49^, ^50^

It has been demonstrated that holoBLG selectively nourishes regulatory immune cells with micronutrients, thereby fostering immune resilience and tolerance.^34^ BLG is a lipocalin, like many of the major allergens, but acts as a tolerogen in context with its ligands (holoBLG) by increasing intracellular iron, importing retinoic acid (RA) via the RA receptor, and activating the arylhydrocarbon receptor via transport of flavonoids such as quercetin, promoting regulatory pathways in a concerted manner.^34^ However, regardless of primarily sensitization against lipocalins or other feline allergens, the supplementation with the lipocalin holoBLG alleviated symptoms in favor of the non‐allergen specific concept of immunonutrition. The primary proof of concept for this antigen‐independent dietary approach was in fact a double blind placebo‐controlled field trial in pollen allergic patients,^37^ demonstrating improved symptoms after nasal allergen provocation by 42% in the holoBLG group (verum) versus 13% in the placebo group. For further proof of concept of the allergen‐nonspecific principle, we subsequently initiated AEC studies, first using house dust mite^38^, ^39^ and finally in the present study cat allergen. In both AEC studies with holoBLG significant symptom improvements could be achieved comparable to the specific effect size in the double‐blind, placebo‐controlled trial in pollen allergic patients.^37^ This strongly suggests that holoBLG‐induced immune dampening pathways in the cat‐allergic patients. To date, we have not yet generated data addressing the long‐term effectiveness of the holoBLG lozenge specifically for cat allergy. However, in a previous study of a similar design in house dust mite‐allergic patients, we demonstrated long‐lasting symptom improvement 7–8 months after cessation of supplementation.^39^ We are tempted to speculate that the allergen‐nonspecific immune memory induced by holoBLG also provides long–term symptom relief for cat allergics.

## 5 CONCLUSION

The results of our study suggest that the management of feline allergic rhinoconjunctivitis by targeted micronutrition with the holoBLG lozenge is feasible, safe and effective. Since the intervention did not include any specific cat allergen, we extrapolated that innate immune mechanisms are underlying its efficacy. In line with previous studies, the allergen‐nonspecific protective farm effect of the holoBLG lozenge can therefore be extended to cat allergy. This study thus offers a novel opportunity in the allergist's tool box for improving the quality of life of cat allergic patients.

## AUTHOR CONTRIBUTIONS

Karl‐Christian Bergmann, Jennifer Raab, Anke Graessel, Sylvia Becker and Matthias F. Kramer designed the study. Data acquisition was done by Karl‐Christian Bergmann, Torsten Zuberbier, Sylvia Becker and Sebastian Kugler. Thomas Zwingers performed the statistical analysis. Karl‐Christian Bergmann, Jennifer Raab, Anke Graessel, Franziska Roth‐Walter, Matthias F. Kramer and Erika Jensen‐Jarolim evaluated and interpreted the data. Karl‐Christian Bergmann, Jennifer Raab, Matthias F. Kramer and Erika Jensen‐Jarolim drafted the manuscript. All authors critically reviewed the manuscript and approved the final version and its submission.

## CONFLICT OF INTEREST STATEMENT

Franziska Roth‐Walter and Erika Jensen‐Jarolim declare inventorship of the patent EP 14150965.3, Year: 01/2014; US 14/204,570 (Method and means for diagnosing and treating allergy; owned by Biomedical International R + D GmbH, Vienna, Austria), underlying the immunoBON® lozenge. Erika Jensen‐Jarolim is a shareholder of Biomedical Int. R + D GmbH, Vienna, Austria and collaborates with Bencard Allergie GmbH in projects and studies on holoBLG. Franziska Roth‐Walter received research funding from Biomedical International R + D GmbH, Vienna, Austria and lecture honoraria by Bencard Allergie GmbH, Munich, Germany and Vienna, Austria, and Allergy Therapeutics, Worthing, UK. Jennifer Raab, Anke Graessel, Thomas Zwingers and Matthias F. Kramer are employees of Allergy Therapeutics/Bencard Allergie GmbH. Karl–Christian Bergmann received honoraria for lectures from ALK, AstraZeneca, Bencard, GSK, HAL, LETI Novartis, Sanofi during last 2 years, works for the German Pollen Information Service Foundation and is member of ECARF‐Institute GmbH. Torsten Zuberbier reports lecture honoraria from AstraZeneca, ALK, AbbVie, Almirall, Astellas, Bayer health Care, Bencard, Berlin Chemie, FAES, HAL, Leti, Meda, menarini, Merck, MSD, Novartis, Pfizer, Sanofi, Stallergenes, Takeda, Teva, UCB, Henkel, Kryolan and LÓreal. Sylvia Becker and Sebastian Kugler report no competing interests.

## CONSENT FOR PUBLICATION

The authors provided their consent for the publication of the study results.

## TRIAL REGISTRATION

The study protocol approved by the Ethics Committee of the Charité Berlin, Germany (EA1/119/21) was retrospectively registered at clinicaltrials.gov (NCT05455749).

## Supporting information

Supporting Information S1

## Data Availability

The datasets generated and/or analyzed during the current study are not publicly available. Bencard Allergie GmbH is committed to providing qualified external researchers with access to pseudonymized patient data and supporting clinical documents from related studies. These requests are reviewed and approved by an independent review panel on the basis of scientific merit.
