# Supplementary material for: The holo beta‐lactoglobulin lozenge reduces symptoms in cat allergy—Evaluation in an allergen exposure chamber and by titrated nasal allergen challenge
Source: Clin Transl Allergy. 2023 Jul 1;13(7):e12274. doi: 10.1002/clt2.12274 (PMC10314279; doi:10.1002/clt2.12274)
Supplement: Supplementary file 1 — Supporting Information S1 [file CLT2-13-e12274-s001.docx]

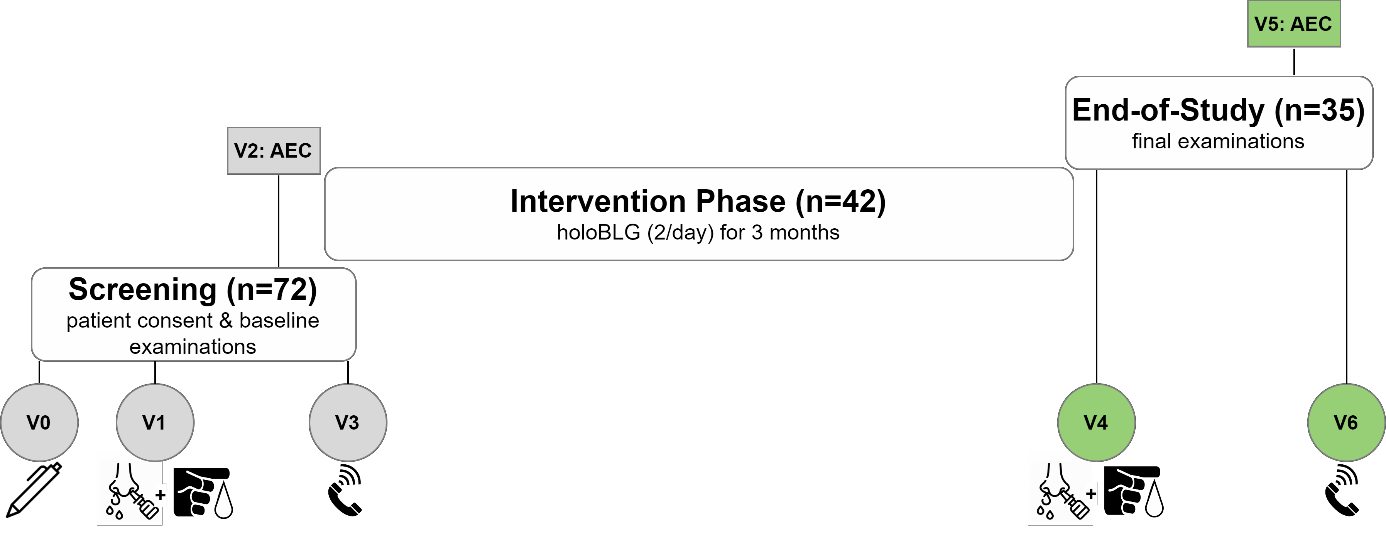


**Supplemental Figure 1** Study design: Visit 0: written informed consent and selection of patients. Visit 1, Visit 4: nasal provocation test and blood sampling approximately 1 week before provocation. Visit 2, Visit 5: cat allergen provocation in the allergen exposure chamber (AEC). Visit 3, Visit 6: Follow-up telephone calls 24 h after provocation.


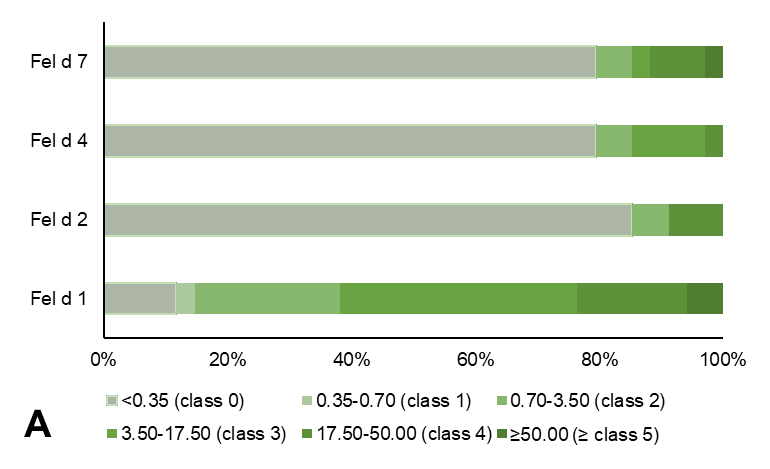


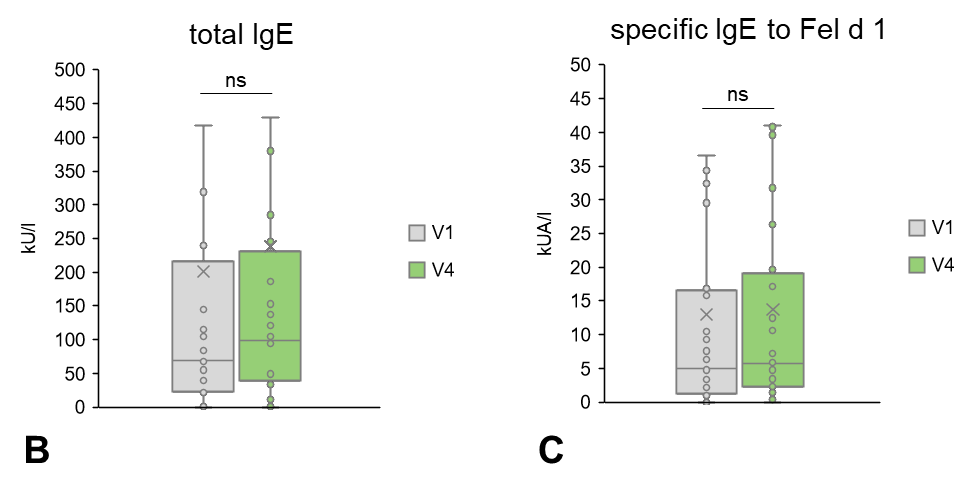


**Supplemental Figure 2** IgE values. A: Sensitization profile of the study population (n=34) at baseline. Depicted are percentages of patients with specific IgE values (IgE classes in kUA/l) against the different cat allergens. B: Total IgE values and specific IgE values to Fel d 1 (C) of the study population (n=34) at baseline (V1) and after intervention (V4). *ns*, not significant
